# Retinal Artery Angles in High Axial Myopia and Its Relationship With Visual Function

**DOI:** 10.1167/tvst.12.8.22

**Published:** 2023-08-29

**Authors:** Jia Liang, Ting Xie, Lu Chen, Canfeng Huang, Pengxue Wei, Pengfeng Li, Ke Liu, Zhenhua Zou, Dong Fang, Shaochong Zhang

**Affiliations:** 1 Shenzhen Eye Hospital, Jinan University, Shenzhen Eye Institute, Shenzhen, Guangdong, China; 2 Guizhou Medical University, Guiyang, Guizhou, China

**Keywords:** high axial myopia, retinal artery angle, scanning laser ophthalmoscopy imaging, visual function

## Abstract

**Purpose:**

To evaluate the retinal artery angles in high axial myopia and assess the correlation with other morphometric and functional parameters.

**Methods:**

This cross-sectional study included 112 eyes of 112 patients with high axial myopia. Based on axial length (AL), the participants were divided into three groups: group 1 (26 ≤ AL < 28 mm), group 2 (28 ≤ AL < 31 mm), and group 3 (≥31 mm). Scanning laser ophthalmoscopy imaging was used to analyze the retinal artery angle (Yugami correlated angle [YCA]). Retinal vascular densities (VDs) in both superficial capillary plexuses (SCPs) and deep capillary plexuses were evaluated. Fixation behavior, including retinal mean sensitivity (MS), macular fovea 2°, 4° fixation rate (P1, P2), and 68.2% bivariate contour ellipse area, were analyzed by microperimetry. Finally, the correlation between YCAs and AL, VDs, best-corrected visual acuity (BCVA), and fixation behavior was assessed.

**Results:**

The YCAs showed significant differences among the three groups (all *P* < 0.001, respectively). Compared to group 1, the YCA decreased in group 2 (*P* < 0.001) and continued to decrease in group 3 (*P* = 0.043). The correlation analysis revealed that smaller YCAs (YCA, YCA_1/2_, YCA_1/4_) were positively correlated with the longer AL (ρ = 0.580, 0.545, 0.448, *P* < 0.001) and lower VDs in any sector in SCPs (all *P* ≤ 0.05). Furthermore, smaller YCAs were positively correlated with decreased BCVA (*ρ* = 0.392, 0.387, 0.262; all *P* < 0.001) and reduced MS (*ρ*= 0.300, 0.269, 0.244; all *P* < 0.05).

**Conclusions:**

Smaller YCAs were correlated with longer AL, lower VD in SCP, decreased BCVA, and reduced MS. The YCAs might reflect vascular deformation caused by axial elongation and could potentially be useful in predicting visual function in high axial myopia.

**Translational Relevance:**

The quantitative analysis of YCAs in fundus photography holds potential clinical value in predicting visual function in high axial myopia.

## Introduction

High myopia (HM) is a prevalent cause of severe visual impairment, with a rising prevalence globally, particularly in East Asian countries.^1^^,^^2^ High axial myopia was defined by an axial length (AL) of 26 mm or greater.^3^ The pathogenesis of high myopia is related to axis length elongation, posterior traction from posterior scleral staphylomas, and lateral traction from vitreoretinal traction. The retinal artery angle refers to the angle between the supratemporal and the infratemporal vascular arches, with the optic disk as the vertex. Previous studies^4^^,^^5^ have utilized the retinal artery angle to evaluate morphologic changes in retinal arteries. The Yugami correlated angle (YCA), a measurement of the retinal artery angle, was first introduced by Nagura et al.^6^ Their study demonstrated that YCAs were significantly smaller in eyes with idiopathic epiretinal membrane (ERM) due to membrane traction. Moreover, it was suggested that YCAs were strongly associated with visual function in eyes with ERM. Hence, YCAs may serve as a valuable parameter for assessing morphologic and functional prognosis in tractional retinal diseases. In individuals with high axial myopia, the retina is subjected to various traction forces resulting from axial length elongation, posterior staphyloma, and vitreomacular traction. Thus, we hypothesized that these traction forces could influence retinal artery angles during the development of high axial myopia.

In addition to axial length changes, high axial myopia frequently leads to alterations in retinal vasculature, including capillary nonperfusion, vascular layer atrophy, and vascular narrowing.^7^ Optical coherence tomographic angiography (OCTA) has been widely employed in the evaluation of retinal vascular diseases due to its noninvasive and high-speed imaging capabilities, allowing visualization of the superficial and deep retinal vasculature and providing detailed information on volumetric blood flow.^8^^,^^9^ While previous studies have identified changes in retinal vascular structure, such as increased foveal avascular zone area (FAZ)^10^ and decreased vessel perfusion in HM with axial length elongation,^11^ the precise mechanisms remain unclear. Furthermore, it is uncertain whether the changes in vessel perfusion correspond to alterations in YCAs. Therefore, a comprehensive investigation of macular vasculature changes in different retinal sectors, with detailed stratification and comparison, is essential to elucidate the underlying pathophysiology of these diseases.

In this study, we aimed to assess alterations of YCAs in high axial myopia and investigate their relationship with axial length, vascular density, and visual function.

## Methods

### Study Patients and Essential Examination

This study was authorized by the Ethics Committee of the Shenzhen eye hospital, Shenzhen, China (2022KYPJ077). All the procedures conformed to the principles of the Declaration of Helsinki. In total, 112 patients aged 18 to 78 years with high axial myopia were enrolled at the Shenzhen eye hospital between April 2021 and May 2022, and all individuals signed informed consent forms. According to AL, the enrolled eyes were assigned into three groups: group 1 (26 ≤ AL < 28 mm), group 2 (28 ≤ AL < 31 mm), and group 3 (≥ 31mm). All participants underwent detailed ophthalmic examinations, including best-corrected visual acuity (BCVA) test, slit-lamp examination, and refractive status assessment using an autorefractor (KR-8800; Topcon, Tokyo, Japan); fixation behavior was analyzed by microperimetry (MP-3; NIDEK Technologies, Aichi, Japan), and the AL was measured using IOL Master 500 (Carl Zeiss Meditec, Jena, Germany). Several studies have reported that myopic eyes have a higher risk of primary open-angle glaucoma.^12^^,^^13^ Therefore, the retinal nerve fiber layer (RNFL) thickness was assessed by the Cirrus HD-OCT (Carl Zeiss Meditec, Dublin, CA, USA). Stereodisc photography (Kowa nonmyd a-D III; Kowa Optimed Inc, Aichi, Japan) and visual field testing (Humphrey Visual Field Analyzer II with SITA standard 24-2; Carl Zeiss Meditec) were performed to rule out glaucoma. Glaucoma was diagnosed based on optic disc changes, including narrowed neuroretinal rim and optic disc excavation with corresponding RNFL abnormalities with or without visual field defects.^14^ Exclusion criteria were as follows: vitreoretinal surgery or refractive surgery history; eyes with keratopathy, cataract, glaucoma, uveitis, retinal detachment, macular retinoschisis or macular hole, macular atrophy, or epiretinal membrane; evidence of retinal vascular diseases, including diabetic retinopathy, age-related macular degeneration, choroidal neovascularization, and use of medication that might affect the vasculature; and images with poor quality, in which it was difficult to recognize the retinal vessels in the macular area.

### OCTA Image Acquisition and Processing

All participants underwent macular (6 × 6 mm) OCTA scans using the Spectral Domain OCT (Optovue, Fremont, CA, USA). This instrument has an A-scan rate of 70 kHz with a light source centered at a wavelength of 840 nm and a bandwidth of 45 nm. The AngioVue software (version 2018.1.1.63; Optovue) was a built-in software that provides a default angiography display protocol to define the stratification interval for en face vascular imaging and automatically calculate the blood flow density. The software automatically segmented the superficial capillary plexuses (SCPs) and deep capillary plexuses (DCPs). The SCP extends from the internal limiting membrane to 10 µm above the inner plexiform layer, and the DCP extends from 10 µm above the inner plexiform layer to the 10 µm below the outer plexiform layer according to the automated setting.

**Figure 1.**
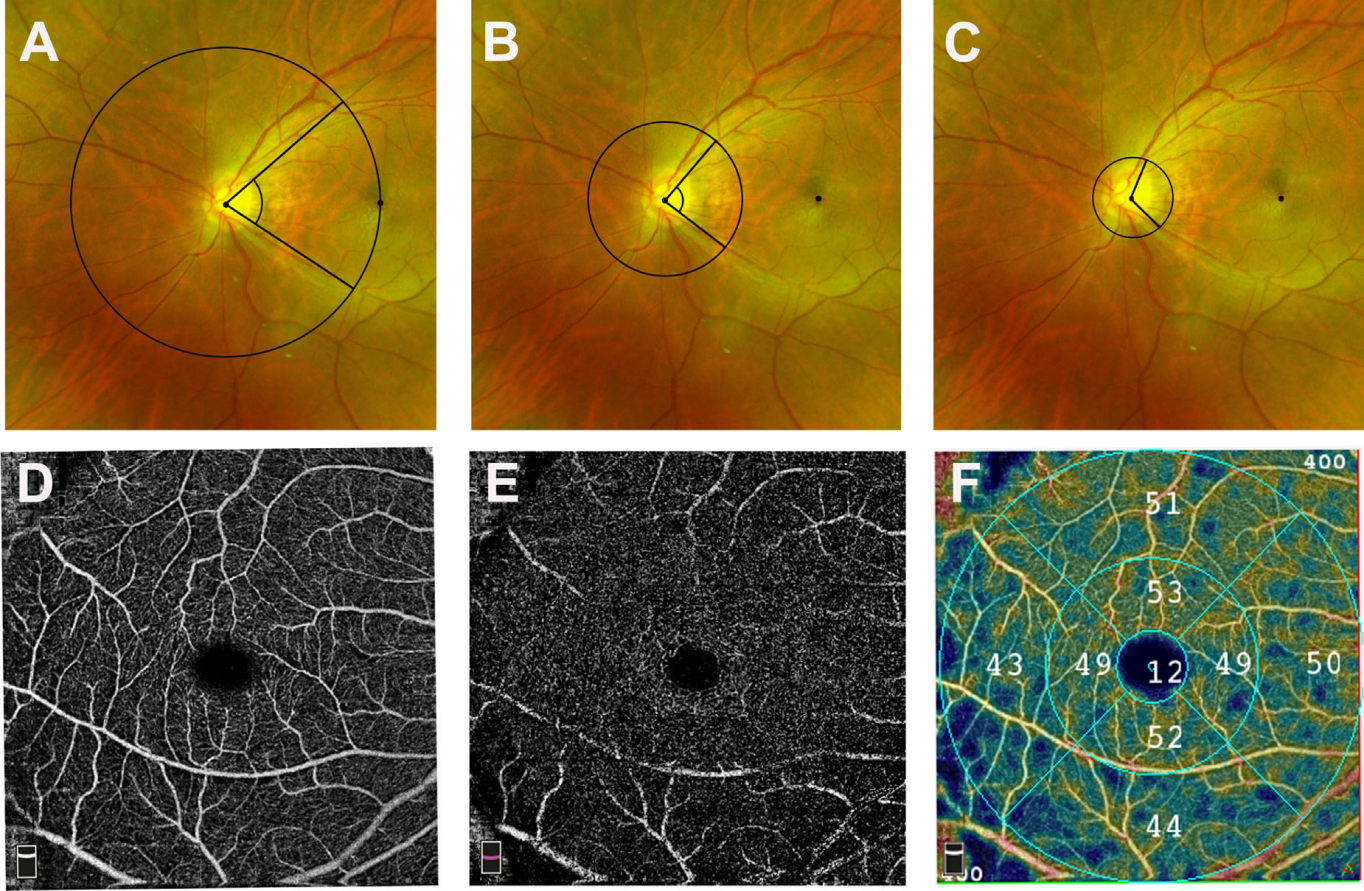
Measurement of Yugami correlated angles (YCA, YCA_1/2_, and YCA_1/4_) and vascular density. (**A**) The YCAs are presented in a scanning laser ophthalmoscopy photograph. A circle was drawn with the radius of the distance between the fovea and optic disc center and the circle crossed the supratemporal and the infratemporal vascular arches. Then, the cross points of the circle were found, and the angle between these cross points and the optic disk center was measured as the YCA. (**B**) The cross points of a half circle were found, and the angle between these cross points and the optic disk center was measured as YCA_1/2_. (**C**) The cross points of a quarter circle were found, and the angle between these points and the optic disk center was measured as YCA_1/4_. (**D**) OCTA scan of superficial retinal plexus. (**E**) OCTA scan of deep retinal plexus. (**F**) Representative 6-mm × 6-mm macular OCTA scans in highly axial myopic eyes. The capillary density is automatically calculated according to the regions of the Early Treatment of Diabetic Retinopathy Study grid. The parafovea is an area with an inner diameter of 1 mm and an outer diameter of 3 mm. The perifovea is an area with an inner diameter of 3 mm and an outer diameter of 6 mm. Parafoveal and perifoveal zones were subdivided into inferior, superior, nasal, and temporal quadrants. Vascular density was corrected by using Bennett’s formula.

The images with a signal intensity score of less than 7 or motion artifacts were excluded.^15^^,^^16^ The OCTA images were corrected for image magnification based on the AL using Bennett’s formula.^17^ The OCTA images were assessed by two proficient ophthalmologists (SCZ and LC) to make sure that the images were eligible. Based on the Early Treatment of Diabetic Retinopathy Study subfields, the macular region was divided into three concentric circles with a diameter of 1, 3, and 6 mm. The inner ring and outer ring were named the parafovea and perifovea, respectively. Further, each ring was subdivided into four quadrants: inferior, superior, nasal, and temporal (Fig. 1). We analyzed the average vascular densities (VDs) of the fovea, parafovea, perifovea, and whole area. Meanwhile, the average VDs in four quadrants of both the parafovea and perifovea were also assessed.

### Scanning Laser Ophthalmoscope Image Acquisition and Processing

The retinal artery angle was photographed using a scanning laser ophthalmoscope (SLO) (Carl Zeiss Meditec). The retinal artery angle was defined as the angle between the supratemporal and the infratemporal vascular arches, with the optic disk as the vertex (named YCA). It was measured by the ImageJ system (version 2.0.0; National Institutes of Health, Bethesda, MD, USA) as suggested by Nagura et al.^6^ For the assessment of the angle YCA, we drew a circle with a radius of the distance between the fovea and optic disk center and determined the intersection points between the supratemporal and infratemporal major retinal artery and the drawn circle. Moreover, the angle between two intersections and the optic disk center in the eyes with high axial myopia was calculated. In addition, we drew circles with a radius of one-half and one-fourth the distance from the fovea to the center of the optic disk. We determined the crossing points between these circles and the arteries of the temporal upper and temporal inferior arcade arteries. The angle between these intersection points and the optic disk center was measured as the YCA_1/2_ and the YCA_1/4_ (Fig. 1).

### Microperimetry Image Acquisition and Processing

A microperimetry examination was performed on patients using MP-3 (NIDEK Technologies) in a light room after pupillary dilation by 1% tropicamide. Goldmann III size stimuli with a duration of 200 ms were used on the background. Background luminance was set at 31.4 asb (10 cd/m^2^), the stimulus’s dynamic range was between 0 and 34 dB, and a 1° diameter red circle was set as a fixation target. A 4–2 staircase-threshold strategy was used to estimate visual sensitivity in the central 20° diameter with 37 stimuli.^18^ Additionally, macular fovea 2°, 4° fixation rate (P1, P2) and 68.2% hyperbolic bivariate contour ellipse area (BCEA) were also used to assess the fixation stability.

### Statistical Analyses

All statistics were analyzed using SPSS 22.0 (SPSS version 22.0; IBM-SPSS, Chicago, IL, USA). Gender differences were compared using the Pearson χ^2^ or Fisher exact test. The BCVA was presented as the logarithm of the minimum angle of resolution. The continuous variables among groups, including age, BCVA, AL, spherical equivalent (SE), mean sensitivity (MS), and VDs, all were skewedly distributed and compared through the Kruskal–Wallis test. YCAs (YCA, YCA_1/2_, YCA_1/4_) were normally distributed and analyzed by a one-way analysis of variance (ANOVA test). Spearman correlation analysis was adopted to test the correlations among AL, VDs, BCVA, and fixation behavior with YCAs. Statistically significant was defined as a *P* value less than 0.05.

## Results

### Clinical Characteristics of Eyes With High Axial Myopia

A total of 112 eyes from 112 patients with high axial myopia were included in the analysis. The study group comprised 63 women and 49 men, with a mean age of 45.7 ± 12.43 years (range, 18.0–78.0). The average BCVA was 0.34 ± 0.44, with a range of −0.18 to 2.00. The mean SE was −9.8 ± 6.0 D, ranging from −6.5 to −27.0 D. The mean AL was 29.21 ± 2.59 mm with a range of 26.00 to 35.73 mm.

Based on the AL, the patients were categorized into three groups: group 1 (*n* = 49, 43.7% of eyes), group 2 (*n* = 28, 25% of eyes), and group 3 (*n* = 35, 31.3% of eyes). The baseline characteristics are presented in detail in Table 1. SE, AL, and BCVA significantly differed among the three groups (all *P* < 0.05, Kruskal–Wallis test). However, no significant difference was observed in age among the three groups (*P* = 0.073, Kruskal–Wallis test). Patients in group 3 had significantly decreased BCVA and lower SE compared to those in group 1 (all *P* < 0.001, Kruskal–Wallis test).

**Table 1.** Patient Characteristics of the Three Study Groups

Characteristic	Group 1 (*n* = 49)	Group 2 (*n* = 28)	Group 3 (*n* = 35)	*P* Value	*P*_1_ Value	*P*_2_ Value	*P*_3_ Value
Sex, female/male	31/18	13/15	19/16				
Age (range), y	44.18 ± 13.42 (18–78)	43.85 ± 14.12 (18–74)	49.31 ± 8.47 (33–70)	0.073	0.911	0.062	0.083
BCVA (range, logMAR)	0.13 ± 0.21 (−0.18 to 1.00)	0.23 ± 0.36 (0.00 to 1.52)	0.69 ± 0.52 (0.00 to 2.00)	**<0.001** ^a^	**0.234**	**<0.001** ^a^	**<0.001** ^a^
Axial length (range), mm	26.87 ± 0.64 (26.00–27.96)	29.15 ± 0.90 (28.07–30.94)	32.53 ± 1.20 (31.07–35.73)	**<0.015** ^a^	**<0.001** ^a^	**<0.001** ^a^	**<0.001** ^a^
SE (range), D	−6.67 ± 2.72 (−11.00 to –6.50)	−10.52 ± 4.87 (−21.25 to –6.50)	−13.51 ± 7.85 (−27.00 to –7.50)	**<0.001** ^a^	**0.001** ^a^	**<0.001** ^a^	**0.029** ^a^

Data are presented as the means ± SD. *P* value among the three groups: *P*_1_ value, *P* value between group 1 and group 2; *P*_2_ value, *P* value between group 1 and group 3; *P*_3_ value, *P* value between group 2 and group 3. Bold values indicate statistical significance *P* < 0.05.

BCVA, best-corrected visual acuity; logMAR, logarithm of the minimum angle of resolution; AL, axial length; SE, spherical equivalent.

a Kruskal–Wallis test.

### YCAs in Patients With Varying Degrees of High Axial Myopia

**Figure 2.**
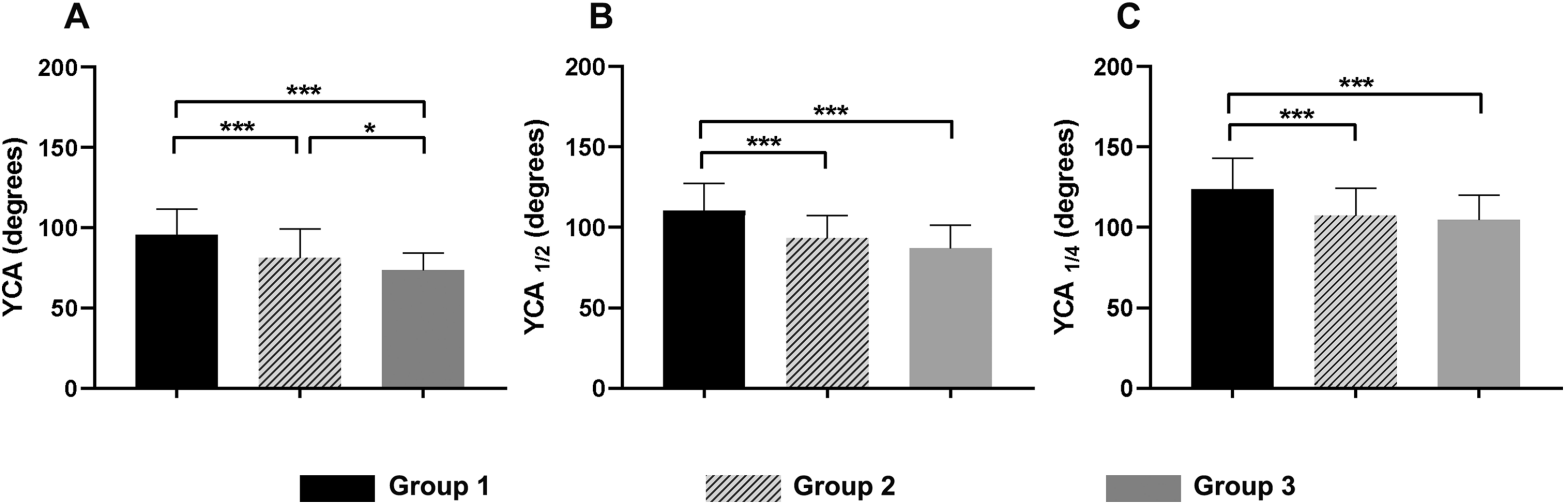
Comparisons of Yugami correlated angles (YCA, YCA_1/2_, and YCA_1/4_) among different high axial myopic groups. YCA showed a downward trend as myopia progressed, and the difference between adjacent groups was statistically significant, as shown in the graphs (**A**). YCA_1/2_ (**B**) and YCA_1/4_ (**C**) decreased in groups 2 and 3 compared with group 1, but no statistical difference was found between the two groups. *P < 0.05, **P ≤ 0.01, ***P ≤ 0.001.

**Figure 3.**
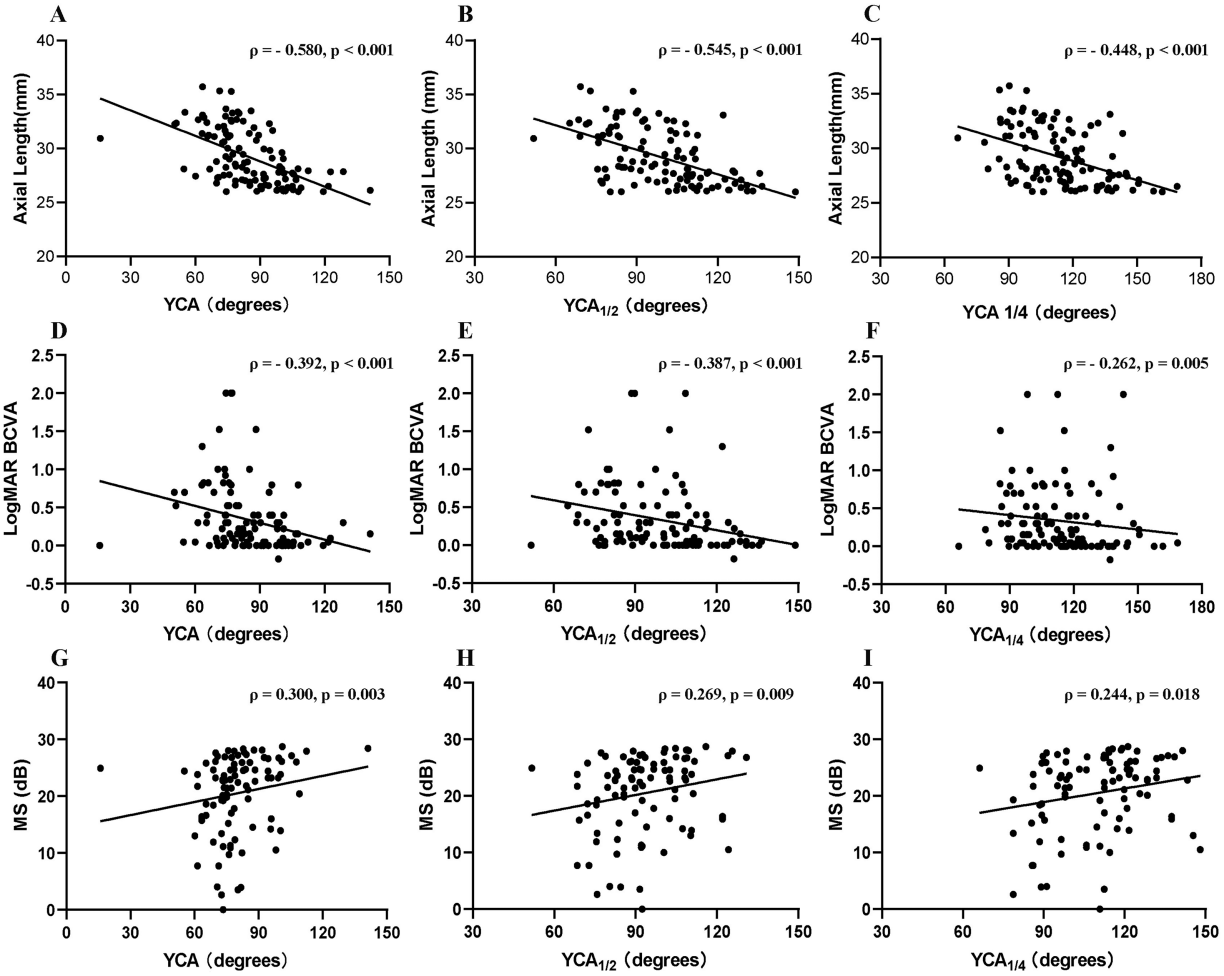
Spearman correlation analysis between AL, logMAR BCVA, and MS with YCAs. AL was negatively correlated with YCA (**A**), YCA_1/2_ (**B**), and YCA_1/4_ (**C**). LogMAR BCVA had a negative correlation with YCA (**D**), YCA_1/2_ (**E**), and YCA_1/4_ (**F**). Conversely, MS was positively associated with YCA (**G**), YCA_1/2_ (**H**), and YCA_1/4_ (**I**). Spearman correlation coefficient (ρ) and *P* value are presented in corresponding graphs. Statistical significance was defined as *P* < 0.05.

For total participants, the mean YCA was 84.97 ± 17.95 (15.93–141.15), the mean YCA_1/2_ was 98.95 ± 18.50 (51.77–148.80), and the mean YCA_1/4_ was 113.66 ± 19.50 (66.22–168.70). Additionally, the average YCA, YCA_1/2_, and YCA_1/4_ in three groups were as follows: 95.39 ± 16.23, 110.53 ± 16.95, and 123.57 ± 19.27 in group 1; 81.27 ± 17.89, 93.42 ± 13.80, and 107.32 ± 16.98 in group 2; and 73.36 ± 10.89, 87.14 ± 14.08, and 104.83 ± 15.26 in group 3 (Table 2 and Fig. 2). Significant differences were found among the three groups regarding YCA, YCA_1/2_, and YCA_1/4_ (all *P* < 0.001, ANOVA test). Two-way comparisons showed that the YCA, YCA_1/2_, and YCA_1/4_ in group 1 were significantly higher than those in group 2 (all *P* < 0.001, ANOVA test) and group 3 (all *P* < 0.001, respectively, ANOVA test). Compared to group 2, group 3 exhibited a smaller YCA (*P* = 0.043, ANOVA test). However, there was no statistically significant difference in YCA_1/2_ and YCA_1/4_ between group 2 and group 3. Furthermore, Spearman correlation analysis demonstrated negative correlations between YCA, YCA_1/2_, and YCA_1/4_ with AL (ρ = −0.580, −0.545, −0.448, *P* < 0.001, respectively) (Table 3 and Fig. 3).

**Table 2.** Comparisons of Yugami Correlated Angles and OCTA Parameters Among High Axial Myopic Groups

Characteristics	Group 1 (*n* = 49)	Group 2 (*n* = 28)	Group 3 (*n* = 35)	*P* Value	*P*_1_ Value	*P*_2_ Value	*P*_3_ Value
YCA	95.39 ± 16.23	81.27 ± 17.89	73.36 ± 10.89	**<0.001** ^a^	**<0.001** ^a^	**<0.001** ^a^	**0.043** ^a^
YCA_1/2_	110.53 ± 16.95	93.42 ± 13.80	87.14 ± 14.08	**<0.001** ^a^	**<0.001** ^a^	**<0.001** ^a^	0.110
YCA_1/4_	123.57 ± 19.27	107.32 ± 16.98	104.83 ± 15.26	**<0.001** ^a^	**<0.001** ^a^	**<0.001** ^a^	0.576
SCP VD—whole, %	46.41 ± 5.99	44.36 ± 6.94	38.29 ± 9.94	**<0.001** ^b^	0.731	**<0.001** ^b^	**0.033** ^b^
SCP VD—parafovea, %	45.35 ± 9.86	44.65 ± 8.60	33.69 ± 11.81	**<0.001** ^b^	1.000	**<0.001** ^b^	**<0.001** ^b^
SCP VD—perifovea, %	48.17 ± 5.68	47.05 ± 6.05	39.67 ± 10.23	**<0.001** ^b^	1.000	**<0.001** ^b^	**0.004** ^b^
SCP VD—fovea, %	19.33 ± 10.08	20.40 ± 11.11	18.11 ± 15.01	0.378	—	—	—
SCP VD—para-T, %	46.33 ± 10.04	44.08 ± 10.31	29.78 ± 12.89	**<0.001** ^b^	0.710	**<0.001** ^b^	**<0.001** ^b^
SCP VD—para-S, %	46.51 ± 11.76	45.35 ± 10.56	32.11 ± 15.48	**<0.001** ^b^	1.000	**<0.001** ^b^	**0.001** ^b^
SCP VD—para-N, %	43.43 ± 10.90	41.01 ± 12.53	35.92 ± 13.73	**0.012** ^b^	1.000	**0.010** ^b^	0.170
SCP VD—para-I, %	45.27 ± 11.39	47.49 ± 8.74	36.61 ± 14.33	**0.002** ^b^	1.000	**0.010** ^b^	**0.005** ^b^
SCP VD—peri-T, %	43.80 ± 5.02	40.88 ± 9.01	33.67 ± 11.08	**<0.001** ^b^	0.654	**<0.001** ^b^	**0.002** ^b^
SCP VD—peri-S, %	49.00 ± 6.41	48.26 ± 7.07	42.12 ± 12.01	**0.011** ^b^	1.000	**0.012** ^b^	0.075
SCP VD—peri-N, %	52.11 ± 6.95	50.44 ± 7.78	47.45 ± 13.77	0.079	—	—	—
SCP VD—peri-I, %	47.99 ± 7.50	45.91 ± 6.83	36.29 ± 11.69	**<0.001** ^b^	0.876	**<0.001** ^b^	**0.001** ^b^
DCP VD—whole, %	43.92 ± 8.38	43.26 ± 10.01	38.61 ± 8.58	**0.006** ^b^	1.000	**0.007** ^b^	**0.040** ^b^
DCP VD—parafovea, %	47.77 ± 10.52	48.09 ± 12.07	40.88 ± 12.37	**0.004** ^b^	1.000	**0.007** ^b^	**0.019** ^b^
DCP VD—perifovea, %	45.33 ± 8.29	44.78 ± 10.15	39.87 ± 9.66	**0.013** ^b^	1.000	**0.016** ^b^	0.070
DCP VD—fovea, %	34.91 ± 13.09	35.97 ± 14.05	27.29 ± 15.88	0.054	1.000	0.058	**0.047** ^b^
DCP VD—para-T, %	49.08 ± 12.17	46.77 ± 16.22	37.92 ± 15.21	**0.001** ^b^	1.000	**0.001** ^b^	**0.022** ^b^
DCP VD—para-S, %	47.29 ± 11.13	49.74 ± 13.73	42.09 ± 16.61	0.064	—	—	—
DCP VD—para-N, %	48.82 ± 11.20	46.74 ± 15.72	40.08 ± 12.39	**<0.001** ^b^	**1.000** ^b^	**<0.001** ^b^	**0.010** ^b^
DCP VD—para-I, %	45.90 ± 10.46	49.29 ± 12.23	43.32 ± 13.68	**0.042** ^b^	0.278	0.852	**0.037** ^b^
DCP VD—peri-T, %	47.35 ± 8.82	44.58 ± 14.29	34.25 ± 13.13	**<0.001** ^b^	1.000	**<0.001** ^b^	**0.002** ^b^
DCP VD—peri-S, %	45.93 ± 8.96	45.48 ± 10.55	42.68 ± 10.88	0.216	—	—	—
DCP VD—peri-N, %	44.11 ± 8.47	43.67 ± 11.82	43.21 ± 10.81	0.892	—	—	—
DCP VD—peri-I, %	43.24± 9.61	44.61 ± 11.14	38.84 ± 11.11	**0.049** ^b^	1.000	0.191	0.059
FAZ, mm^2^	0.39 ± 0.81	0.35 ± 0.49	0.83 ± 1.82	0.051^b^	1.000	0.117	0.085

Data are presented as the means ± SD. *P* value among the three groups: *P*_1_ value, *P* value between group 1 and group 2; *P*_2_ value, *P* value between group 1 and group 3; *P*_3_ value, *P* value between group 2 and group 3. Bold values indicate statistical significance *P* < 0.05.

para-I, parafoveal inferior; para-N, parafoveal nasal; para-S, parafoveal superior; para-T, parafoveal temporal; peri-I, perifoveal inferior; peri-N, perifoveal nasal; peri-S, perifoveal superior; peri-T, perifoveal temporal.

a ANOVA test.

b Kruskal–Wallis test.

**Table 3.** Spearman Correlation Between Axial Length, the Retinal Vascular Density, and Visual Function With the Yugami Correlated Angles

	YCA		YCA_1/2_		YCA_1/4_	
Characteristics	ρ	*P* Value	ρ	*P* Value	ρ	*P* Value
AL	−0.580	<**0.001**	−0.545	<**0.001**	−0.448	<**0.001**
SCP-VD—whole, %	0.435	<**0.001**	0.423	<**0.001**	0.322	**0.001**
SCP-VD—parafovea, %	0.447	<**0.001**	0.441	<**0.001**	0.344	<**0.001**
SCP-VD—perifovea, %	0.397	<**0.001**	0.387	<**0.001**	0.283	**0.003**
SCP-VD—fovea, %	0.068	0.477	0.066	0.492	0.081	0.397
SCP-VD—para-T, %	0.434	<**0.001**	0.405	<**0.001**	0.287	**0.002**
SCP-VD—para-S, %	0.403	<**0.001**	0.392	<**0.001**	0.287	**0.002**
SCP-VD—para-N, %	0.327	**0.005**	0.355	<**0.001**	0.318	**0.001**
SCP-VD—para-I, %	0.299	**0.008**	0.279	<**0.001**	0.195	<**0.001**
SCP-VD—peri-T, %	0.427	<**0.001**	0.414	<**0.001**	0.334	<**0.001**
SCP-VD—peri-S, %	0.330	**0.015**	0.325	<**0.001**	0.302	<**0.001**
SCP-VD—peri-N, %	0.335	**0.043**	0.320	<**0.001**	0.239	<**0.001**
SCP-VD—peri-I, %	0.414	<**0.001**	0.402	<**0.001**	0.301	<**0.001**
DCP-VD—whole, %	0.331	**0.003**	0.249	<**0.001**	0.186	<**0.001**
DCP-VD—parafovea, %	0.337	<**0.001**	0.252	**0.007**	0.162	0.088
DCP-VD—perifovea, %	0.300	**0.001**	0.223	**0.018**	0.172	0.07
DCP-VD—fovea, %	0.173	0.068	0.118	0.216	0.162	0.089
DCP-VD—para-T, %	0.310	**0.001**	0.268	**0.004**	0.239	**0.011**
DCP-VD—para-S, %	0.217	**0.021**	0.113	0.237	0.038	0.688
DCP-VD—para-N, %	0.396	<**0.001**	0.330	<**0.001**	0.246	**0.009**
DCP-VD—para-I, %	0.144	0.13	0.083	0.382	0.067	0.483
DCP-VD—peri-T, %	0.382	<**0.001**	0.320	**0.001**	0.238	**0.011**
DCP-VD—peri-S, %	0.216	**0.022**	0.14	0.14	0.084	0.38
DCP-VD—peri-N, %	0.146	0.124	0.083	0.382	0.043	0.651
DCP-VD—peri-I, %	0.157	0.099	0.084	0.380	0.101	0.289
FAZ, mm^2^	−0.073	0.442	−0.026	0.787	−0.094	0.323
BCVA, logMAR	−0.392	<**0.001**	−0.387	<**0.001**	−0.262	<**0.001**
MS, dB	0.300	**0.003**	0.269	**0.009**	0.244	**0.018**
P1, %	0.101	0.331	0.151	0.146	0.119	0.254
P2, %	0.126	0.226	0.156	0.134	0.125	0.229
68.2% BCEA, deg^2^	−0.129	0.216	−0.186	0.073	−0.135	0.195

Bold values indicate statistical significance *P* < 0.05.

### Vascular Densities in High Axial Myopia and Their Relationship With YCAs

**Figure 4.**
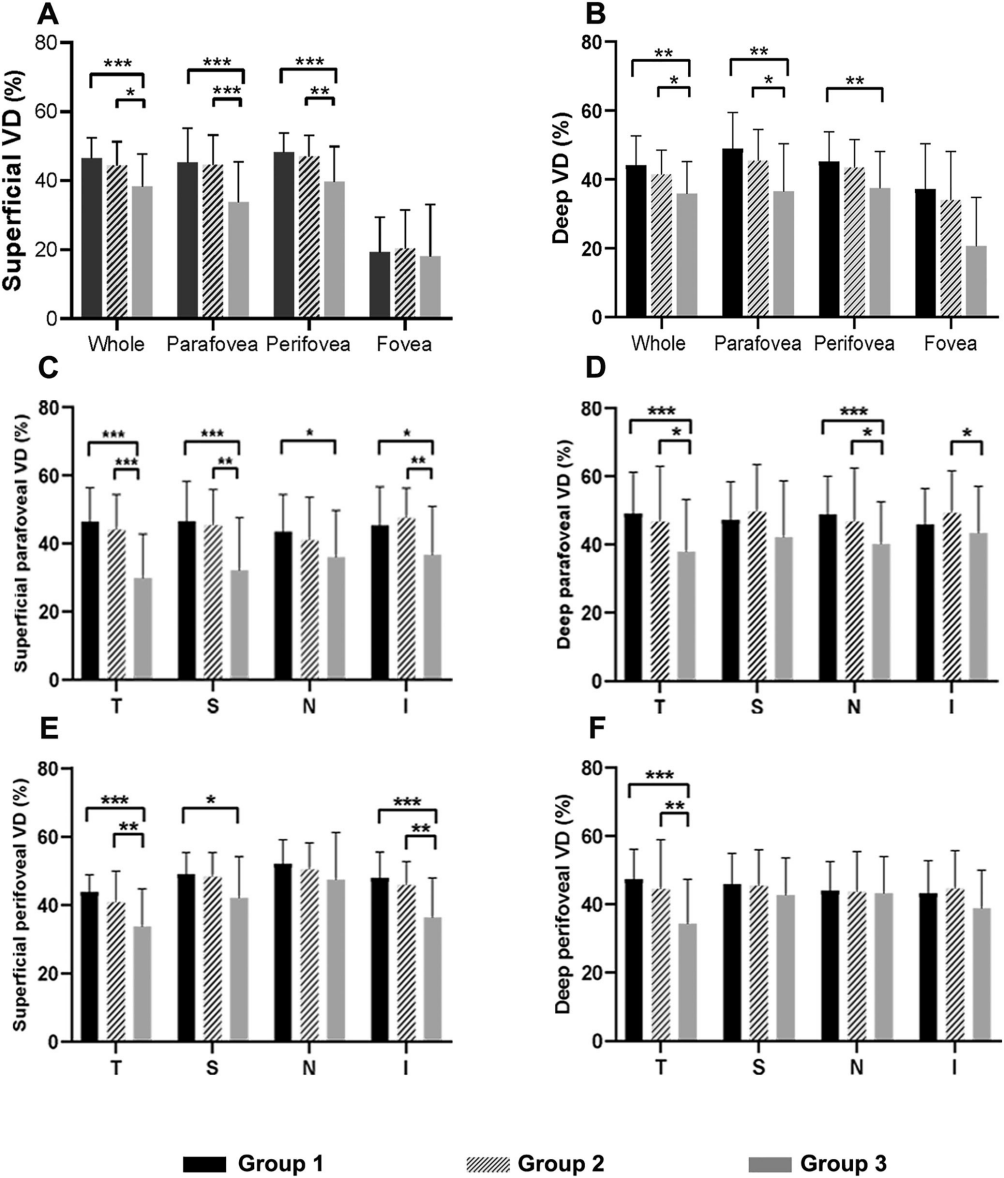
Comparisons of vascular densities (%) in different zones among three high axial myopic groups. Superficial (**A**) and deep (**B**) retinal layers generally presented lower microvascular densities in whole, parafoveal, and perifoveal zones in groups with the group 3, except for the foveal zone. As myopia groups changed from the group 1 to group 3, vascular densities decreased unevenly in four quadrants in both retinal layers (**C**–**F**). *P* value among the three groups: P_1_, *P* value between group 1 and group 2; *P_2_*, *P* value between group 1 and group 3; *P*_3_, *P* value between group 2 and group 3. *P < 0.05, **P ≤ 0.01, ***P ≤ 0.001.

The VDs of the SCP and DCP in different macular zones were assessed (Table 2 and Fig. 4). Compared to group 1, the whole, parafoveal, and perifoveal VDs in group 3 were significantly reduced in both SCP and DCP (all *P* < 0.05, Kruskal–Wallis test). Similarly, compared to group 2, the group 3 showed significantly lower VDs in the whole and parafoveal zone in both retinal microvascular layers (*P* = 0.033, <0.001 in SCP; *P* = 0.040, 0.019 in DCP, Kruskal–Wallis test) as well as the superficial perifoveal zone (*P* = 0.004, Kruskal–Wallis test). However, there were no significant differences in the foveal VDs among three groups in both retinal layers (*P* = 0.378 in SCP, *P* = 0.054 in DCP, Kruskal–Wallis test) (Table 2 and Fig. 4).

Generally, the VD values over the four quadrantal partitions were significantly different among the three groups in both SCP and DCP, except for that in perifoveal nasal quadrants in both plexuses (*P* = 0.079 in SCP, *P* = 0.892 in DCP, Kruskal–Wallis test), as well as in perifoveal superior quadrant and parafoveal superior quadrant in DCP (*P* = 0.216, 0.064, Kruskal–Wallis test) (Table 2 and Fig. 4). Compared to group 1, VDs in group 2 did not lessen in perifoveal and parafoveal temporal sectors within both SCP and DCP as well as parafoveal superior and perifoveal inferior sectors in SCP (all *P* > 0.05, Kruskal–Wallis test). However, they decreased significantly in group 3 (all *P* ≤ 0.001, Kruskal–Wallis test). Additionally, no significant differences were observed in the FAZ among the three groups (*P* = 0.051, Kruskal–Wallis test) (Table 2 and Fig. 4).

As regards their relationship with YCAs, positive correlations were found with all YCAs in any sector in SCP (all *P* ≤ 0.05). Especially in perifoveal, perifoveal temporal and parafoveal superior zones, VDs presented a moderate positive correlation with YCAs (YCA, YCA_1/2_, YCA_1/4_) (ρ = 0.447, 0.441, 0.344 and all *P* < 0.001 for the perifoveal zone; ρ = 0.427, 0.414, 0.334 and all *P* < 0.001 for the perifoveal temporal zone; ρ = 0.403, 0.392, 0.287 and all *P* < 0.01 for the parafoveal superior zone) in SCP. Besides, all YCAs correlated positively with VDs in the parafoveal temporal (ρ = 0.310, 0.268, 0.239 and all *P* < 0.05), parafoveal nasal (ρ = 0.396, 0.330, 0.246, and all *P* < 0.01), and perifoveal temporal (ρ = 0.382, 0.320, 0.238 and all *P* < 0.05) sectors in DCP. However, no correlations were found between YCAs and foveal VDs in either SCP or DCP and the FAZ (all *P* > 0.05) (Table 3).

### Correlation Between YCAs and Visual Function

The average BCVA was 0.34 ± 0.44 with a range of –0.18 to 2.00. For each subgroup, BCVA was as follows: 0.13 ± 0.21 in group 1, 0.23 ± 0.36 in group 2, and 0.69 ± 0.52 in group 3. The MS was 20.45 ± 6.95 dB, ranging from 0.00 to 28.70 dB. MS in the three groups was 25.11± 5.61 dB, 19.93 ± 7.50 dB, and 18.94 ± 5.94 dB. The mean fixation rates P1 and P2 were 75.06% ± 24.00% and 90.56% ± 13.28%. Moreover, the mean 68.2% BCEA was 4.06 ± 5.1 deg^2^. Smaller YCA, YCA_1/2_, and YCA_1/4_ were significantly positively associated with decreased BCVA (ρ = 0.392, 0.387, 0.262; all *P* < 0.001) and reduced MS (ρ = 0.300, 0.269, 0.244; all *P* < 0.05). However, no significant correlations were found between YCAs and P1, P2, and 68.2% BCEA (Table 3 and Fig. 3).

## Discussion

High axial myopia is characterized by elongation of the eyeball and is associated with various pathologic mechanisms, including traction forces from the elongated AL, posterior staphyloma, and vitreomacular traction. In this study, we investigated the YCAs as a parameter related to retinal traction in high axial myopia. We found that YCAs decreased significantly with increasing AL in high axial myopia, and they were associated with retinal VDs in the SCP, BCVA, and retinal MS. This suggests that the measurement of the retinal artery angle was useful for predicting both the degree of retinal traction and visual function in patients with high axial myopia. To our knowledge, this study is the first to examine the alteration of YCAs and their significance in evaluating high axial myopia.

Our results are partially in accordance with previous studies that reported a decrease in retinal artery angles with AL elongation in HM. Jonas et al.^4^ documented a reduction in retinal artery angles in eyes with high axial myopia, which correlated with AL elongation. Similarly, Jonas et al.^5^ found a more frequent reduction in retinal artery angles in eyes with HM compared to those without HM during a 10-year follow-up, attributed to AL elongation. In this study, we obtained similar outcomes, demonstrating that retinal artery angles decreased with AL elongation in HM. However, the methods used to measure retinal artery angles differed between our study and theirs. They used the angle kappa, which involves confirming the crossing points of the temporal superior and inferior arterial arcade with a vertical line passing through the fovea and then forming the angle kappa between these crossing points and the center of the optic disc. In contrast, we determined YCAs using three circles with the optic disc as the center, with each circle’s radius corresponding to specific distances between the optic disc and the fovea. This utilization of three vascular angle indices provided by YCA allows for an accurate description of the vascular trajectory at different distances from the optic disc, leading to a more precise understanding of vascular characteristics. Our results showed a continuous downward trend of YCA from group 1 to group 3, which is consistent with the findings of Jonas et al.^4^ This may be explained by the longitudinal stretching of the retina rather than its widthwise expansion due to AL elongation. Furthermore, previous research has mathematically explained the decrease in retinal artery angle resulting from the elongation of the disc–fovea distance (DFD) and the constant vertical distance between the artery arcades.^5^ Interestingly, a longer AL is associated with a longer DFD, indicating that AL elongation may be the primary cause of a decreased retinal artery angle. Several magnetic resonance imaging studies^19^^,^^20^ have revealed that myopic eyes exhibit increased dimensions in all three axes, particularly in length (0.35 mm/D) compared to height (0.19 mm/D) and width (0.10 mm/D). This supports our speculation that the retina primarily stretches in the axial direction rather than widthwise due to the proximity of the orbital walls to the sides of the eyes compared to behind the eyes.^20^ However, YCA_1/2_ and YCA_1/4_ have no significant differences between the latter groups. This could be because the angle was closer to the optic disc, resulting in lower susceptibility to AL elongation.

By evaluating the VDs in high axial myopia using OCTA, we found a decrease in VDs in the whole, parafoveal, and perifoveal areas, with the parafoveal region showing a more significant reduction compared to the perifoveal zones. The result was in line with Shi et al.,^21^ who showed that VDs decrease closer to the fovea. The thinness of the fovea and its surrounding parafoveal region may make it more susceptible to elongation during AL elongation in high axial myopia. On the contrary, Li et al.^22^ reported no significant difference in VDs between the parafoveal and perifoveal regions. The inconsistent result may be attributed to variations in sample sizes and grouping principles. Furthermore, we found no significant differences in foveal VDs and the area of the FAZ among the three groups, which is consistent with previous studies.^23^^,^^24^ This can be attributed to the inherently low VDs in the foveal region due to the presence of FAZ. When it comes to sectoral alterations of macular VD, our statistical result suggested that group 3 presented more significant changes in macular VDs in the parafoveal temporal and perifoveal temporal sectors in both retinal capillary plexuses and parafoveal, parafoveal superior, and perifoveal inferior sectors in SCP compared to the other two groups. However, there were no differences in VDs in these sectors between group 1 and group 2. These outcomes indicated that these sectors might be crucial areas with higher sensitivity to detect capillaries in high axial myopia. In contrast, the change of VDs had no significant difference in the perifoveal nasal sector among the three groups, which partially agrees with the study by Liu et al.,^25^ suggesting that the perifoveal nasal sector may have relatively lower susceptibility to AL elongation. A possible explanation for this result is that the sector may have abundant blood perfusion due to the presence of large retinal vascular branches, as identified by OCTA with an 8-mm^2^ × 8-mm^2^ macular region scanning.^26^

We analyzed the association among AL, VDs in all sectors, BCVA, and fixation behavior with YCAs. We found a significant negative correlation between YCAs and AL, suggesting that the elongation of eyeballs had a remarkable impact on the morphology of retinal vessels, which is in line with the previous reports.^5^ Additionally, YCAs were correlated with VDs in any sector in SCP. The changes of macular VDs in these sectors were correlated with the YCAs, indicating that YCAs may be an indicator reflecting the VD loss. Presumably, the change of retinal artery angle is influenced by various traction forces, such as AL elongation and eyeball expansion. Our hypothesis aligns with the studies^6^^,^^27^ suggesting that retinal vessels become straightened and attenuated, leading to decreased vascular perfusion due to axial elongation and eyeball expansion in high myopia. In other words, straightened retinal vessels led to a change in YCAs, accompanied by a decrease in VDs. Another possible explanation is that the straightening of retinal vessels might result from the stretching papilla–macular nerve fibers and thinning of the RNFL. Omoto et al.^28^ revealed that the RNFL thickness decreased significantly with the narrowing of the retinal artery angle, which may affect regional oxygen demand or the need for vascular supply, thereby causing the loss of VDs.^27^ Additionally, smaller YCAs were significantly associated with decreased BCVA and reduced MS. We postulated that smaller YCAs, accompanied by longer AL and macular dystrophy, result in decreased BCVA and MS. This hypothesis is supported by Chiang et al.,^29^ who demonstrated that the patients with macular dystrophy had worse BCVA, foveal MS, and fixation MS and poorer stability compared to control groups. Therefore, we inferred that YCAs might be a crucial indicator for assessing alterations in VDs and visual function.

This study has several limitations. First, this study is cross-sectional research, which prevents us from determining causality between the retinal vascular densities and retinal artery angles, as well as the dynamic changes of retinal vasculature during the progression of highly axial myopia. Second, the small sample size, especially in group 2 and group 3, may affect the reliability of our results. However, our sample size was adequate to provide a detection power of over 95% based on sample size calculation with G*power (version 3.1.9). Third, only good-quality OCTA and SLO images were included in the analysis, which may introduce selection bias and impact the generalizability of the outcomes.

## Conclusions

In conclusion, we found a significant reduction in YCAs in high axial myopia as the axial length increased. Furthermore, YCAs are closely correlated with VDs and visual function in eyes with high axial myopia. The change in retinal artery angles may result from axial elongation and vitreoretinal traction, providing valuable insights to understand the underlying pathophysiologic mechanisms of myopia progression. However, further longitudinal research is needed to investigate the relationships between retinal structure, function, and retinal artery angles.
